# Leiomyosarcoma of the small bowel presenting as an acute small bowel obstruction

**DOI:** 10.1093/jscr/rjae419

**Published:** 2024-06-24

**Authors:** Erika Wilt, Grant McDaniel, Jennifer Stiene, Madison Stolly, Rongjun Guo, David Diep

**Affiliations:** College of Medicine, University of Toledo College of Medicine and Life Sciences, 3000 Arlington Avenue, Toledo, OH 43614 United States; College of Medicine, University of Toledo College of Medicine and Life Sciences, 3000 Arlington Avenue, Toledo, OH 43614 United States; College of Medicine, University of Toledo College of Medicine and Life Sciences, 3000 Arlington Avenue, Toledo, OH 43614 United States; College of Medicine, University of Toledo College of Medicine and Life Sciences, 3000 Arlington Avenue, Toledo, OH 43614 United States; Promedica Pathology, Consultants in Laboratory Medicine, 2130 West Central Avenue, Toledo, OH 43606 United States; Promedica Physicians General Surgery, Adrian Charles and Virginia Hickman Hospital, 5640 North Adrian Highway, Adrian, MI 49221 United States

**Keywords:** small intestine tumor, leiomyosarcoma, ileal tumor, sarcoma

## Abstract

Leiomyosarcoma is a subtype of soft-tissue sarcoma, which is a rare soft-tissue malignancy comprising < 1% of adult cancers. There are a variety of etiologies of small bowel obstruction. Infrequently, small bowel malignancies can first present as small bowel obstruction. In exceedingly rare cases, leiomyosarcomas can be the offending malignancy. A 53-year-old male presented to the emergency department with several weeks of persistent right abdominal pain, nausea, and vomiting. Computed tomography scan revealed a central necrotic mass within the right lower quadrant originating from the small bowel. The patient underwent exploratory laparotomy to relieve the obstruction and a mass was identified originating from the terminal ileum that adhered to surrounding structures. Pathological analysis determined the mass to be small bowel leiomyosarcoma. Leiomyosarcoma is definitively diagnosed after primary resection with histopathology and immunohistochemistry. As opposed to other small bowel neoplasms, surgical resection with negative margins is the only potentially curative option.

## Introduction

Primary small bowel tumors are rare. When identified they are often comprised of adenocarcinoma, neuroendocrine tumors, gastrointestinal stromal tumors (GIST) and sarcomas [1, 2]. Sarcomas comprise < 1% of all adult cancer diagnoses, leiomyosarcomas constituting 20%–30% of those cases [2, 3]. Leiomyosarcomas originate from the embryonic mesoderm and in exceedingly rare cases can lead to a small bowel malignancy. The following case report describes a patient presenting with a malignant small bowel obstruction that, after surgical resection and pathologic evaluation, was identified as leiomyosarcoma.

## Case report

A 53-year-old male presented to the emergency department (ED) with complaints of persistent right sided abdominal pain, nausea, and vomiting for several weeks. He was initially seen at an Urgent Care and was diagnosed with gastroenteritis. His past medical and surgical history was significant for type two diabetes mellitus, history of deep vein thrombosis anticoagulated with aspirin 325 mg daily, horseshoe kidney status post right nephrectomy, and gout. In the ED his vitals were heart rate 100 bpm, respiratory rate 20, blood pressure 113/75, SpO_2_ 99%, and temperature of 37.5 C. Physical exam was significant for abdominal tenderness in the right upper and lower quadrants, negative for costovertebral angle tenderness bilaterally, and negative for peritoneal signs. The ensuing work up showed a positive fecal occult blood test and abdominal X-ray showing air-filled loops of small bowel measuring 4.8 cm in cross-section, concerning for small bowel obstruction. There was no free air and a moderate colonic stool burden. A computed tomography (CT) abdomen was ordered to further characterize the small bowel obstruction revealing a necrotic mass in the right lower quadrant. The surgical team was consulted and the patient was admitted for treatment.

**Figure 1:**
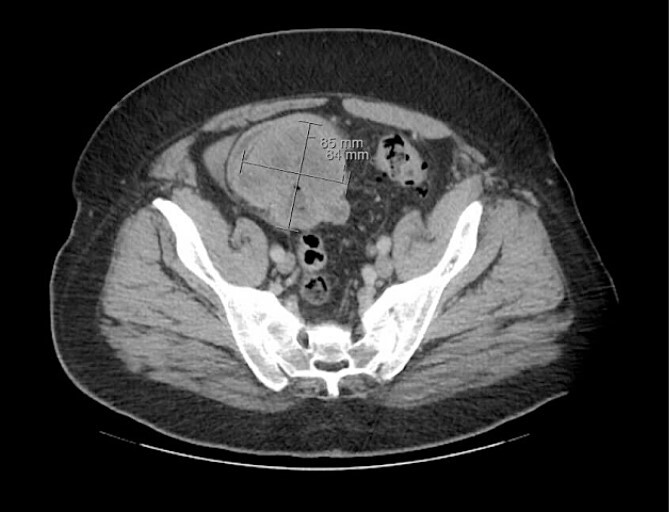
Initial CT imaging, axial cut, showing necrotic mass causing small bowel obstruction.

**Figure 2:**
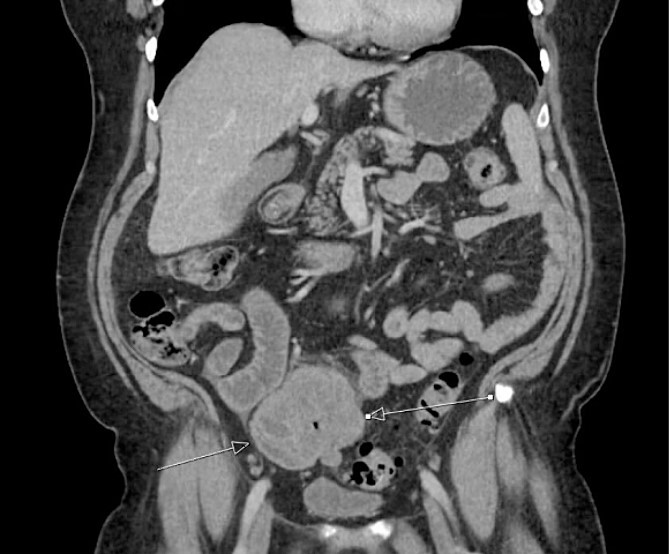
Initial CT imaging, coronal cut, showing necrotic mass causing small bowel obstruction.

**Figure 3:**
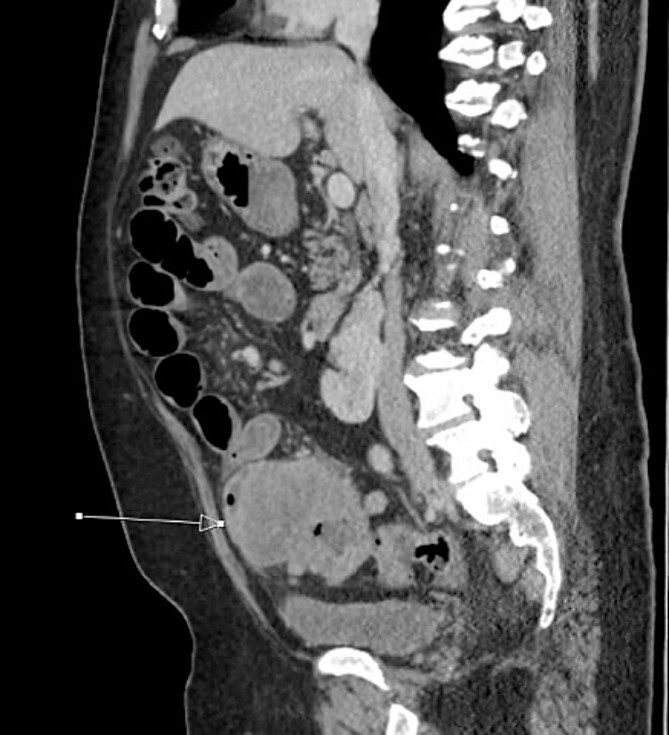
Initial CT imaging, sagittal cut, showing necrotic mass causing small bowel obstruction.

The patient was initially managed nonoperatively with conservative measures including nasogastric (NG) tube decompression, pain control, and serial abdominal exams. Surgery was consulted once CT imaging was obtained (Figs 1–3) and recommended that the patient undergo surgery to resect the identified mass and relieve the obstruction. During the surgical exploration, a mass originating from the terminal ilium was identified that was densely adhered to the peritoneum, bladder, and sigmoid colon, requiring careful dissection. The mass, three lymph nodes, and 29 cm of associated small bowel segment was resected with grossly negative margins, with the mass measuring 7 cm × 8 cm × 9.5 cm. A side-to-side anastomosis was created and the specimens were sent to pathology for further investigation. Postoperatively the patient was hospitalized for 7 days for pain control and monitoring. On postoperative day eight he was discharged with follow up appointments with oncology, general surgery, and a referral to an academic cancer center.

**Figure 4:**
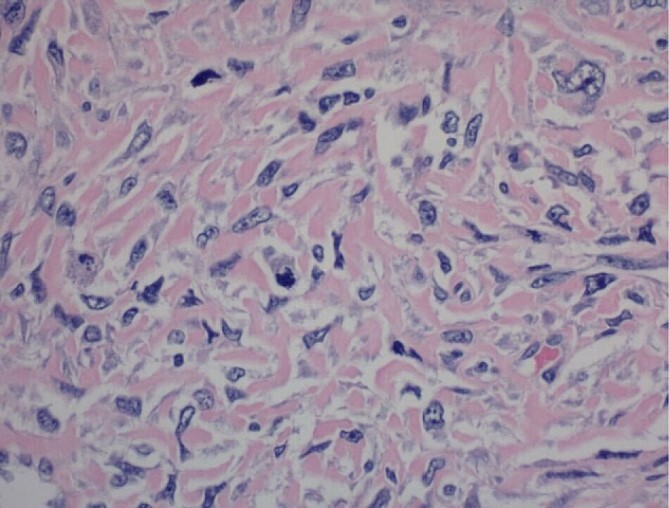
400× magnification. Hematoxylin and eosin stain of tumor cells showing mitotic figures.

**Figure 5:**
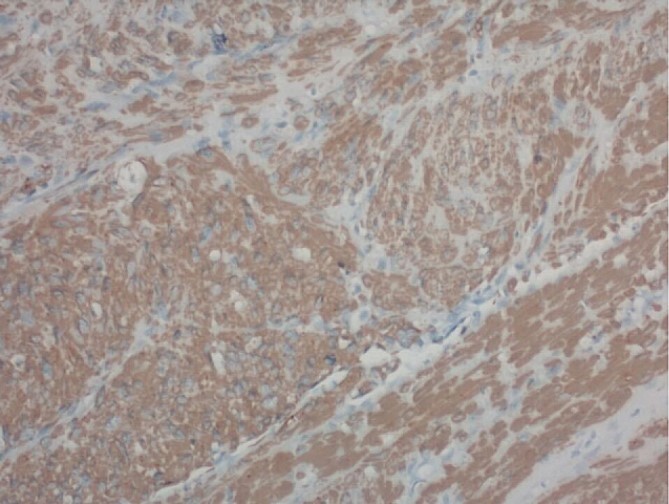
200× magnification of tumor cells. Immunohistochemical stain for smooth muscle actin is positive.

**Figure 6:**
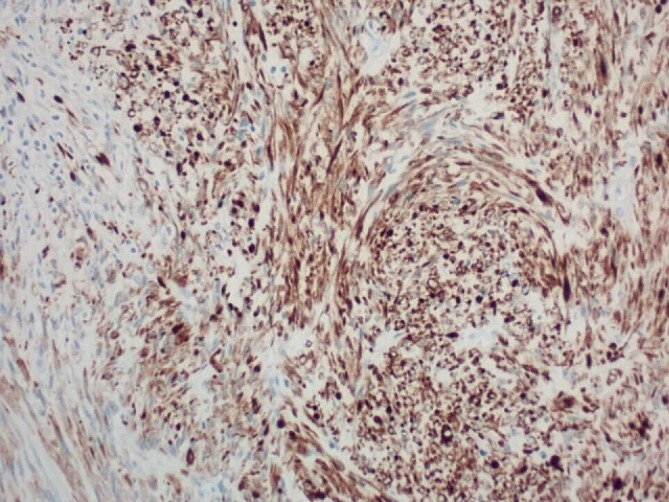
200× magnification of tumor cells. Immunohistochemical stain for desmin is positive.

Pathology report was significant for mitotic figures on hematoxylin and eosin stain (Fig. 4). Immunohistochemistry showed actin positivity (Fig. 5) and desmin positivity (Fig. 6) in the tumor cells. All lymph nodes and margins were negative, with the smallest margin measuring 1.5 cm. The findings listed in the pathology report were consistent with primary leiomyosarcoma of the small bowel.

This case was discussed at a multidisciplinary tumor board meeting at an academic cancer center. The pathology and immunohistochemical staining was repeated to confirm the diagnosis and the sarcoma board recommended adjuvant doxorubicin/ifosfamide if the final pathology supported a chemotherapy sensitive sarcoma subtype. Close follow up was recommended due to the high risk of recurrence and/or metastasis from the primary tumor. Follow-up CT abdomen and pelvis was completed eight weeks after discharge and showed evidence of local recurrence or metastatic disease in the right anterior abdomen with a new 2 cm by 1.7 cm soft tissue nodule separate from the small bowel. There was indeterminate nonenlarged right lower quadrant mesenteric lymph nodes reported that were significant when compared to the initial CT scan.

## Discussion

We report a case of primary ileal leiomyosarcoma that presented acutely as a malignant small bowel obstruction. Leiomyosarcoma is a rare small bowel malignancy that often presents as chronic nonspecific symptoms including abdominal pain, melena, or chronic anemia. It is not routinely identified on upper or lower endoscopy, making it hard to screen for and commonly missed, likely contributing to the later stage of presentation [4].

With the changes of the classification system for gastrointestinal tumors in the mid-1990s and the emergence of definitive tools to differentiate gastrointestinal stromal tumors (GIST) and leiomyosarcoma, primary leiomyosarcomas have become a rare and uncommon diagnosis [5]. Most mesenchymal tumors of the small bowel are classified as carcinoids, adenocarcinoma, lymphomas, GIST, and leiomyosarcomas [4].

It is extremely difficult to differentiate between leiomyosarcoma and GIST, as many of the tumors present uniquely and symptoms and appearance of the tumor can vary [6]. The tumors are definitively diagnosed with histopathology and immunohistochemistry, with leiomyosarcoma showing actin and desmin positive reactivity consistent with smooth muscle cell origin and CD117, CD34, PDGFRA, and DOG1 negativity [3].

Accurate diagnosis and differentiation from other gastrointestinal tumors is crucial as the only curative treatment for leiomyosarcoma is complete resection with clear surgical margins. Surgical resection is only indicated for localized cases, and up to 40% of patients will develop recurrent local or metastatic disease after excision [7]. The accepted first-line therapy for metastatic and otherwise unresectable leiomyosarcoma is gemcitabine or anthracycline based therapies which offer an overall survival time of 14–16 months [7].

## Conclusions

This case presents a 53-year-old man who presented with a malignant small bowel obstruction that was post-operatively diagnosed as a leiomyosarcoma. Leiomyosarcomas are a rare subtype of sarcomas that can be misdiagnosed as GIST or adenocarcinoma of the small bowel. As with many bowel malignancies the presenting symptoms are often vague and nonspecific including abdominal pain, fatigue, anemia, and changes in bowel habits. In patients presenting with these symptoms, it is important to have a high index of suspicion for malignancy. Leiomyosarcoma can only be definitively diagnosed after primary resection and histopathology and immunohistochemistry. If negative margins are not obtained or there is metastasis, palliative chemotherapy can be used to alleviate symptoms and extend survival. As opposed to other small bowel neoplasms, surgical resection with negative margins is currently the only potentially curative option for leiomyosarcoma.
